# Safety and Effect of 12‐Month Ecopipam Treatment in Pediatric Patients with Tourette Syndrome

**DOI:** 10.1002/mdc3.70091

**Published:** 2025-05-12

**Authors:** Donald L. Gilbert, David J.B. Kim, Meredith M. Miller, Sarah D. Atkinson, George B. Karkanias, Frederick E. Munschauer, Stephen P. Wanaski, Timothy M. Cunniff

**Affiliations:** ^1^ Division of Neurology, Cincinnati Children's Hospital Medical Center Department of Pediatrics, University of Cincinnati College of Medicine Cincinnati Ohio USA; ^2^ Emalex Biosciences Chicago Illinois USA; ^3^ Paragon Biosciences Chicago Illinois USA

**Keywords:** dopamine, dopamine D1 receptor, movement disorders, tic disorder, Tourette

## Abstract

**Background:**

Tourette syndrome (TS) is a chronic neurodevelopmental tic disorder with a considerable quality of life (QOL) burden.

**Objectives:**

The goal was to determine the long‐term safety, tolerability, and clinical effects of ecopipam, a first‐in‐class dopamine D1 receptor antagonist, for TS.

**Methods:**

This 12‐month, open‐label extension (OLE) study enrolled patients age ≥6 to ≤18 years with confirmed TS who completed a phase 2b randomized, placebo‐controlled, 12‐week trial. Ecopipam was titrated over 4 weeks to achieve a target oral dose of 1.8 mg/kg/day. Study visits occurred at baseline, monthly for 12 months, and 7 and 14 days after last dose.

**Results:**

A total of 121 patients were included (74% male; 68% age 12–18 years), and 80 (66%) completed the study. Ecopipam was well tolerated. The most common adverse events were nasopharyngitis (14.0%) and anxiety (9.1%). At month 12, there were no significant changes from baseline in body mass index Z‐score (mean [standard deviation] change, 0.05 [0.43]; *P* = 0.35), glycated hemoglobin (0.03% [0.31]; *P* = 0.60), or total cholesterol (0.2 mmol/L [0.7]; *P* = 0.14). No notable changes in scales assessing akathisia, movement disorders, anxiety, or depression occurred. At all time points, significant improvements (*P* < 0.001 vs. baseline) in both the Yale Global Tic Severity Scale Total Tic Score and the Gilles de la Tourette Syndrome Quality of Life Scale for Children and Adolescents total score were observed.

**Conclusions:**

Twelve months of ecopipam dosing was well tolerated during this study and no new adverse events were detected. Compared to baseline, significantly reduced TS symptom severity and improved QOL were observed in children and adolescents.

Tourette syndrome (TS) is a chronic neurodevelopmental disorder that begins in childhood and is characterized by motor and vocal tics of varying frequency, severity, and complexity.^1^ In addition to tics, patients with TS frequently present with comorbid behavioral and emotional disorders (eg, attention deficit hyperactivity disorder [ADHD], obsessive‐compulsive disorder [OCD]).^2^, ^3^, ^4^ Together, these conditions predispose patients to difficulties with socializing, poor self‐perception, impaired performance at school, and, consequently, reduced quality of life (QOL).^2^, ^4^, ^5^ Approximately 1.4 million individuals in the United States have TS or another persistent tic disorder; among children and adolescents age 3 to 17 years, the estimated TS prevalence is 0.2% to 0.3%, with higher rates in males.^6^, ^7^

Treatment options for patients with TS differ according to the severity and complexity of symptoms and include behavioral and/or pharmacologic therapies.^1^ Current clinical practice guidelines recommend pharmacotherapies when comprehensive behavioral intervention for tics is not available or inadequately controls tics, and when the benefits of medication use outweigh the potential safety risks.^1^, ^8^, ^9^ The antipsychotics aripiprazole, haloperidol, and pimozide target the dopamine D2 receptor and are approved in the United States for the treatment of TS.^10^ However, these medications are associated with adverse effects such as weight gain, dyslipidemia, hyperglycemia, prolonged QT interval, somnolence, anhedonia, elevated prolactin levels, and development of movement disorders (eg, akathisia, dystonia, withdrawal dyskinesia, tardive dyskinesia).^8^, ^9^, ^10^, ^11^, ^12^ In addition, α‐2 adrenergic (α2) receptor agonists (eg, guanfacine, clonidine), which are frequently used therapies for pediatric patients with TS, are associated with adverse cardiac effects, including bradycardia, hypotension, and QT prolongation.^13^, ^14^ Furthermore, a randomized, placebo‐controlled trial (N = 34) did not find a meaningful effect of guanfacine for tic reduction in a pediatric population with a chronic tic disorder.^15^

Ecopipam is a first‐in‐class selective dopamine D1 receptor (D1R) antagonist under investigation as a potential treatment for TS.^16^ In a phase 2b, randomized, double‐blind, placebo‐controlled trial of 153 patients age ≥6 to <18 years with TS, treatment with ecopipam tablets (1.8 mg/kg/day for 12 weeks) significantly improved Yale Global Tic Severity Scale Total Tic Score (YGTSS‐TTS) and Clinical Global Impression of Tourette Syndrome Severity (CGI‐TS‐S) compared with placebo.^16^ Significant and clinically meaningful improvements in TS symptoms were observed without evidence of the cardiac or metabolic complications associated with currently available therapies.^16^ Furthermore, no drug‐induced movement disorders were reported during the 12‐week study, and mean changes from baseline in weight and QT interval were similar for ecopipam and placebo.^16^

Given the need for long‐term data on the administration of ecopipam, a 12‐month open‐label extension (OLE) study was conducted to evaluate the long‐term safety, tolerability, and durability of effect of ecopipam in pediatric patients with TS who completed the phase 2b, randomized, placebo‐controlled trial.^16^

## Methods

### Study Design and Patient Population

This was a multicenter, 12‐month OLE (ClinicalTrials.gov identifier NCT04114539) of a phase 2b double‐blind, placebo‐controlled trial.^16^ Patients age ≥6 to ≤18 years with confirmed TS who completed the phase 2b trial through the 14‐day follow‐up visit (after titration off the study drug)^16^ within the previous 30 days were eligible for inclusion. Key exclusion criteria were a history of other neurologic conditions associated with abnormal movements; history of schizophrenia, bipolar disorder, or other psychotic disorders; any unstable primary mood disorder (according to *Diagnostic and Statistical Manual of Mental Disorders, Fifth Edition* criteria) at baseline; major depressive episode in the previous 2 years; history of attempted suicide; and medications that would have unfavorable interactions with ecopipam, including those that affect dopamine signaling (eg, bupropion, tetrabenazine, monoamine oxidase inhibitors, and St. John's wort). Stable doses of concomitant medications prescribed for the treatment of ADHD, including psychomotor stimulants and depressants, were permitted.

The study protocol, including informed consent and assent forms, was approved by the institutional review board at each study center. The study was conducted in accordance with the International Council on Harmonization of Good Clinical Practice and the ethical principles of the Declaration of Helsinki. For patients age <18 years, parents or legal guardians provided written informed consent and patients provided written assent before the initiation of any study procedures. Patients age 18 years independently provided written informed consent.

**FIG. 1:**
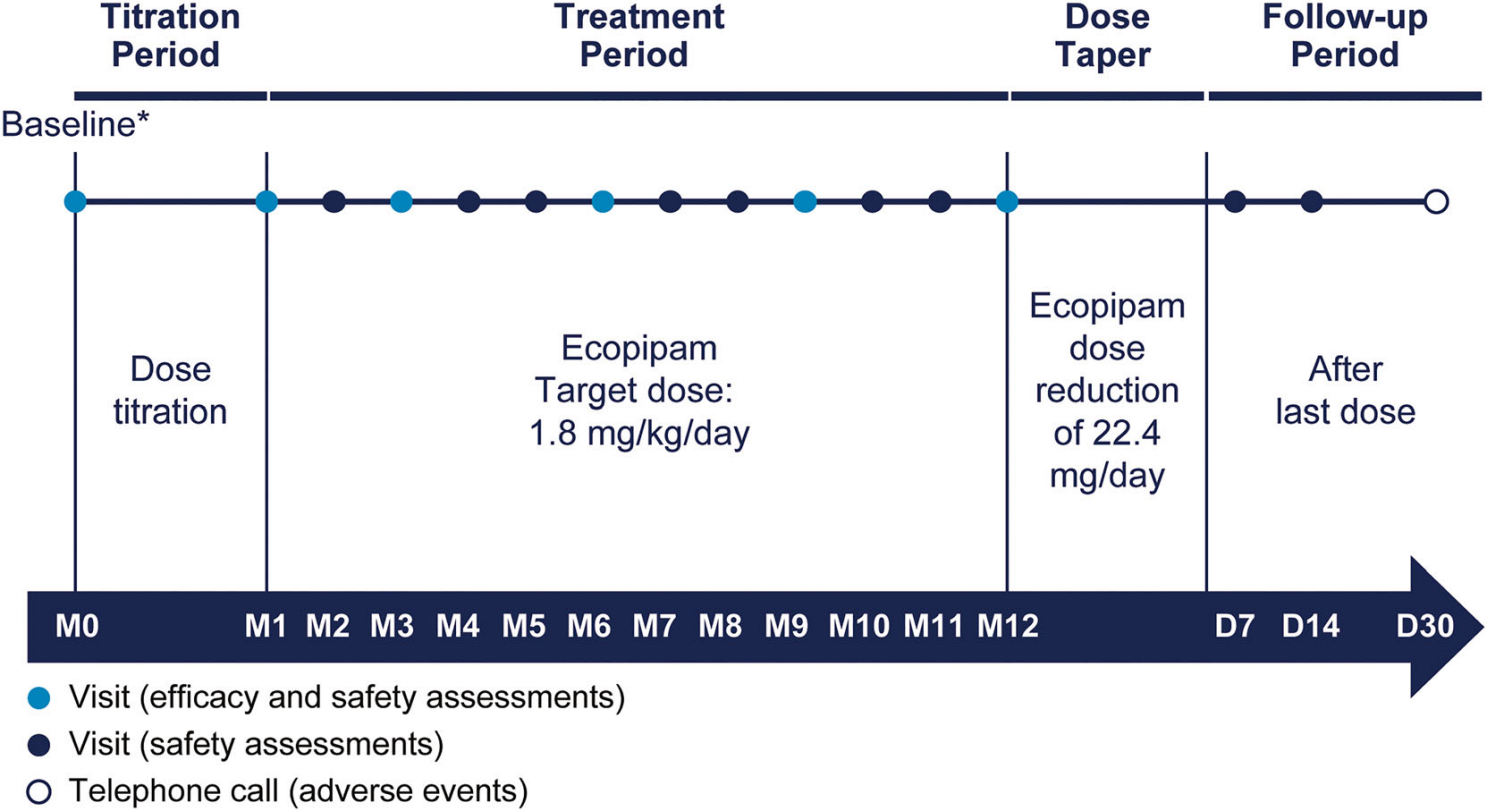
Study design. *Within 30 days of the phase 2b study 14‐day follow‐up visit. D, Day; M, Month.

Ecopipam was titrated over a 4‐week period to achieve a once‐daily target oral dose of 1.8 mg/kg/day (Fig. 1). Dosing during the treatment period (from 4 weeks onward) was based on body weight categories: ≥18 to ≤23 kg (33.6 mg ecopipam), >23 to ≤34 kg (44.8 mg ecopipam), >34 to ≤44 kg (67.2 mg ecopipam), >44 to ≤68 kg (89.6 mg ecopipam), >68 to ≤83 kg (134.4 mg ecopipam), and >83 kg (179.2 mg ecopipam). Dosing adjustments for tolerability were permitted at the discretion of the investigator. At the end of the 12‐month treatment period, the ecopipam dose was tapered by 22.4 mg/day until completely discontinued.

### Assessments

Study visits were conducted at baseline (month 0), monthly (±3 days) for 12 months (until end of the treatment period or early termination), and during two follow‐up visits at 7 (±3) and 14 (±3) days after receipt of the last dose of ecopipam (Fig. 1). In addition, a follow‐up telephone call was conducted 30 (± 3) days after the last dose of ecopipam. Vital signs were recorded at each visit. Physical examinations and laboratory testing were conducted at baseline; months 1, 3, 6, 9, and 12 (or early termination); and the two follow‐up visits. Electrocardiograms (ECGs) were conducted at the same time points except follow‐up visits. Adverse events (AEs) were recorded according to the Medical Dictionary for Regulatory Activities (version 25.0) at each study visit, including the 30‐day follow‐up telephone call; patients were counted once for multiple incidents of the same reported AE. Several AEs of special interest (eg, including events that have been associated with dopamine modulation) were prespecified (see Data S1). The Columbia‐Suicide Severity Rating Scale (C‐SSRS), Abnormal Involuntary Movement Scale (AIMS), Barnes Akathisia Rating Scale (BARS), Children's Depression Rating Scale‐Revised (CDRS‐R), and Pediatric Anxiety Rating Scale (PARS) were assessed at baseline and monthly for 12 months (until end of the treatment period or early termination) to evaluate suicidal ideation and behavior, tardive dyskinesia, drug‐induced akathisia, signs and symptoms of depression, and symptoms of anxiety, respectively. The C‐SSRS was also assessed at the two follow‐up visits.

Durability of effect of ecopipam was assessed by the YGTSS‐TTS, YGTSS‐TTS motor and vocal subscales, YGTSS Impairment (YGTSS‐I), YGTSS Global Score (YGTSS‐GS), CGI‐TS‐S, Clinical Global Impression of Tourette Syndrome Improvement (CGI‐TS‐I), and Gilles de la Tourette Syndrome Quality of Life Scale for Children and Adolescents (C&A‐GTS‐QOL) at baseline (with the exception of CGI‐TS‐I) and months 1, 3, 6, 9, and 12 (or early termination).

### Statistical Analysis

The safety population included all patients who received ≥1 dose of study drug, and the modified intent‐to‐treat population included all patients who received ≥1 dose of study drug and had ≥1 post‐baseline YGTSS scoring. Statistical analyses were performed using SAS 9.4 or higher (SAS Institute, Cary, NC). Safety outcomes were summarized using descriptive statistics. Changes from baseline in YGTSS, CGI‐TS‐S, and C&A‐GTS‐QOL were evaluated using a paired *t* test. CGI‐TS‐I was summarized by visit. Body mass index (BMI), height, and weight were transformed into Z‐scores using age‐ and gender‐matched reference data from the Centers for Disease Control and Prevention National Center for Health Statistics. Post‐hoc analyses of mean changes from OLE baseline to month 12 in metabolic parameters were conducted using a paired *t* test. Treatment adherence was calculated as follows: (number of days drug was taken, divided by number of days in which study drug was to be taken) × 100.

## Results

### Patients

Among 124 patients enrolled at 39 centers in Canada, Poland, and the United States, 121 (97.6%) received ≥1 dose of ecopipam and 121 had ≥1 post‐baseline YGTSS scoring. Approximately three‐quarters of the 121 patients (73.6%) were male and approximately two‐thirds (67.8%) were 12 to 18 years of age (Table 1). Eighty patients (66.1%) completed the study. The most common reasons for study discontinuation were withdrawal of consent/withdrawal by parent/caregiver (n = 18) or AEs (n = 14) (Table S1). Three patients discontinued from the trial during the dose titration period. The mean (standard deviation [SD]) adherence rate during the study was 94.3% (12.9), and duration of exposure was 271.6 (109.8) days, with the majority of patients (66.9%) exposed to ecopipam for ≥10 months.

**TABLE 1:** Patient baseline demographic and disease characteristics

Characteristic	Overall OLE (n = 121)	Received ecopipam in RCT (n = 58)	Received placebo in RCT (n = 63)
Age, mean (SD), y	12.8 (2.8)	12.9 (2.9)	12.8 (2.8)
Age group, n (%)			
6–11 y	39 (32.2)	17 (29.3)	22 (34.9)
12–18 y	82 (67.8)	41 (70.7)	41 (65.1)
Male, n (%)	89 (73.6)	43 (74.1)	46 (73.0)
Race, n (%)			
White	110 (90.9)	51 (87.9)	59 (93.7)
Black	7 (5.8)	5 (8.6)	2 (3.2)
Asian	3 (2.5)	1 (1.7)	2 (3.2)
Other	1 (0.8)	1 (1.7)	0
Hispanic or Latino, n (%)	14 (11.6)	7 (12.1)	7 (11.1)
Medical history, n (%)			
ADHD	54 (44.6)	26 (44.8)	28 (44.4)
Anxiety disorder	28 (23.1)	15 (25.9)	13 (20.6)
OCD	18 (14.9)	10 (17.2)	8 (12.7)
Depression	6 (5.0)	3 (5.2)	3 (4.8)
Weight, mean (SD), kg	56.7 (21.5)	58.1 (23.3)	55.4 (19.9)
YGTSS‐TTS score, mean (SD)^a^	29.6 (8.2)	28.1 (8.3)	31.1 (7.9)
YGTSS‐I score, mean (SD)^a^	27.1 (9.9)	26.0 (10.1)	28.1 (9.6)
YGTSS‐GS score, mean (SD)^b^	56.7 (16.3)	54.1 (17.0)	59.2 (15.4)
CGI‐TS‐S score, mean (SD)^c^	4.3 (0.9)	4.2 (0.9)	4.5 (1.0)
C&A‐GTS‐QOL total score, mean (SD)^d^	26.6 (16.5)^e^	27.4 (17.2)	26.0 (16.0)

^a^ Range from 0 (“none”) to 50 (“severe”).

^b^ Range from 0 to 100 (worse).

^c^ Range from 1 (normal, “not ill at all”) to 7 (“among the most extremely ill”).

^d^ Range from 0 (no problem) to 108 (extreme problem).

^e^ n = 120.

Abbreviations: OLE, open‐label extension; RCT, randomized controlled trial; SD, standard deviation; ADHD, attention deficit hyperactivity disorder; OCD, obsessive‐compulsive disorder; YGTSS‐TTS, Yale Global Tic Severity Scale Total Tic Score; YGTSS‐I, Yale Global Tic Severity Scale Impairment; YGTSS‐GS, Yale Global Tic Severity Scale Global Score; CGI‐TS‐S, Clinical Global Impression of Tourette Syndrome Severity; C&A‐GTS‐QOL, Gilles de la Tourette Syndrome Quality of Life Scale for Children and Adolescents.

### Safety and Tolerability

Ecopipam was well tolerated, and the most common AEs were nasopharyngitis (14.0%) and anxiety (9.1%) (Table 2). For the 69.4% of patients who experienced ≥1 AE, most had a maximum AE intensity of mild or moderate. Serious AEs were reported in two patients, one of which (obsessive thoughts) was considered possibly related to treatment. Among the 14 patients (11.6%) who discontinued because of an AE, depression or related terms (n = 4 [28.6%]) and anxiety (n = 2 [14.3%]) were the most common reasons (Table S2). Among 40 patients with treatment‐related AEs, the most commonly reported were anxiety (n = 8 [6.6%]), depression or related terms (n = 7 [5.8%]), insomnia (n = 7 [5.8%]), and somnolence (n = 7 [5.8%]). AEs of special interest were reported in 24 of 121 patients (19.8%) (Table S3). Those reported in three or more patients were depression or related terms (n = 9 [7.4%]), pyrexia (n = 7 [5.8%]), and suicidal ideation (n = 3 [2.5%]). Dose changes were not deemed necessary by the investigators in the three patients with suicidal ideation. Dose reductions because of an AE occurred in six patients (100 mg to 75 mg [n = 3]; not determined [n = 2]; 75 mg to 12.5 mg [n = 1]), all of whom received placebo during the phase 2b trial. Metabolic parameters were comparable across study time points (Table 3). No significant changes from baseline were observed at month 12 for glycated hemoglobin (mean [SD] change, 0.03% [0.31]; *P* = 0.60), total cholesterol (0.2 [0.7] mmol/L; *P* = 0.14), triglycerides (−0.09 [0.64] mmol/L; *P* = 0.46), diastolic blood pressure (0.9 [10.0] mm Hg; *P* = 0.44), or systolic blood pressure (0.3 [11.5] mm Hg; *P* = 0.85). No substantial changes from baseline in other vital signs or ECG measurements were observed during the study. In addition, there was no significant change from baseline at month 12 in BMI Z‐score (mean [SD] change, 0.05 [0.43]; *P* = 0.35), and height and weight Z‐scores indicated that pediatric patients were maintaining a healthy growth curve (data not shown).

**TABLE 2:** Summary of AEs

Patients with an AE	Patients (n = 121), No. (%)
Any AE	84 (69.4)
Treatment‐related AE^a^	40 (33.1)
Discontinuation due to AE	14 (11.6)
Severe AE	6 (5.0)
Serious AE^b^	2 (1.7)
Most common AEs^c^	
Nasopharyngitis	17 (14.0)
Anxiety	11 (9.1)
Depression^d^	9 (7.4)
Diarrhea	9 (7.4)
Headache	9 (7.4)
Insomnia	9 (7.4)
Somnolence	8 (6.6)
Pyrexia	7 (5.8)
Upper respiratory tract infection	7 (5.8)
COVID‐19	6 (5.0)
Nausea	6 (5.0)
Upper abdominal pain	6 (5.0)
Decreased appetite	5 (4.1)
Tic	5 (4.1)
Vomiting	5 (4.1)
Most common treatment‐related AEs^c^	
Anxiety	8 (6.6)
Depression^e^	7 (5.8)
Insomnia	7 (5.8)
Somnolence	7 (5.8)

^a^ AEs with relationship to treatment as “possibly related” or “probably related” or missing.

^b^ Obsessive thoughts, possibly related to treatment and resulting in treatment withdrawal (1 patient) and serious accidental injury with AEs not related to treatment (1 patient).

^c^ Reported in ≥4.0% of patients.

^d^ Included depression, depressed mood, depressive symptom, and major depression.

^e^ Included depression, depressed mood, and depressive symptom.

Abbreviations: AE, adverse event; COVID‐19, coronavirus disease 2019.

**TABLE 3:** Metabolic parameters

Parameter	OLE baseline	Month 3	Month 6	Month 12
BMI, mean (SD), kg/m^2^	n = 121 22.1 (5.9)	n = 105 22.3 (6.2)	n = 93 23.3 (13.1)	n = 75 22.1 (5.4)
HbA1c, mean (SD), %	n = 57 5.4 (0.3)	n = 94 5.3 (0.3)	n = 82 5.3 (0.3)	n = 69 5.4 (0.3)
Total cholesterol, mean (SD), mmol/L	n = 59 3.9 (0.7)	n = 94 3.9 (0.6)	n = 84 4.0 (0.7)	n = 72 4.1 (0.8)
Triglycerides, mean (SD), mmol/L	n = 59 1.2 (0.7)	n = 94 1.0 (0.5)	n = 84 1.1 (0.5)	n = 72 1.1 (0.6)
Blood pressure, mean (SD), mm Hg	n = 114	n = 98	n = 91	n = 75
Diastolic	69.1 (8.5)	68.6 (8.2)	68.7 (9.4)	70.6 (8.6)
Systolic	112.9 (10.6)	113.4 (10.9)	111.4 (11.4)	113.7 (8.7)

Abbreviations: OLE, open‐label extension; BMI, body mass index; SD, standard deviation; HbA1c, glycated hemoglobin.

Additional safety assessments, including AIMS (presence and severity of movement disorders involving the extremities, face, mouth, and trunk), BARS (drug‐induced akathisia), CDRS‐R (signs and symptoms of depression), and PARS (severity of anxiety symptoms), showed no notable changes from baseline during 12 months of treatment (Table S4). As noted above, suicidal ideation was reported as an AE in three patients. When C‐SSRS data were evaluated, suicidal ideation was noted for ≤5% of patients over all time points. No suicidal behavior was identified during the study.

### Durability of Effect

**FIG. 2:**
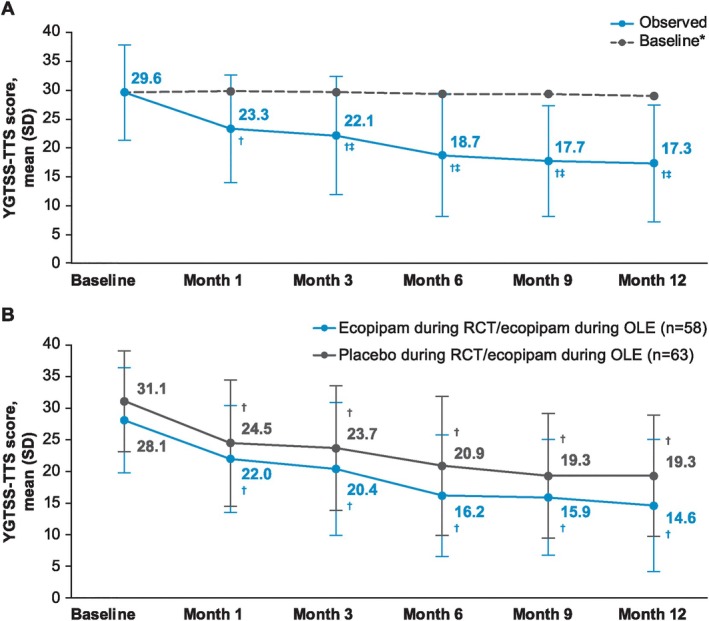
TS severity on the YGTSS‐TTS (**A**) overall and (**B**) by RCT arm, with lower scores indicating improvement.*Baseline for patients with data at that time point. ^†^ *P* < 0.0001 vs baseline. ^‡^≥25% reduction from baseline. OLE, open‐label extension; RCT, randomized controlled trial; SD, standard deviation; TS, Tourette syndrome; YGTSS‐TTS, Yale Global Tic Severity Scale Total Tic Score.

**FIG. 3:**
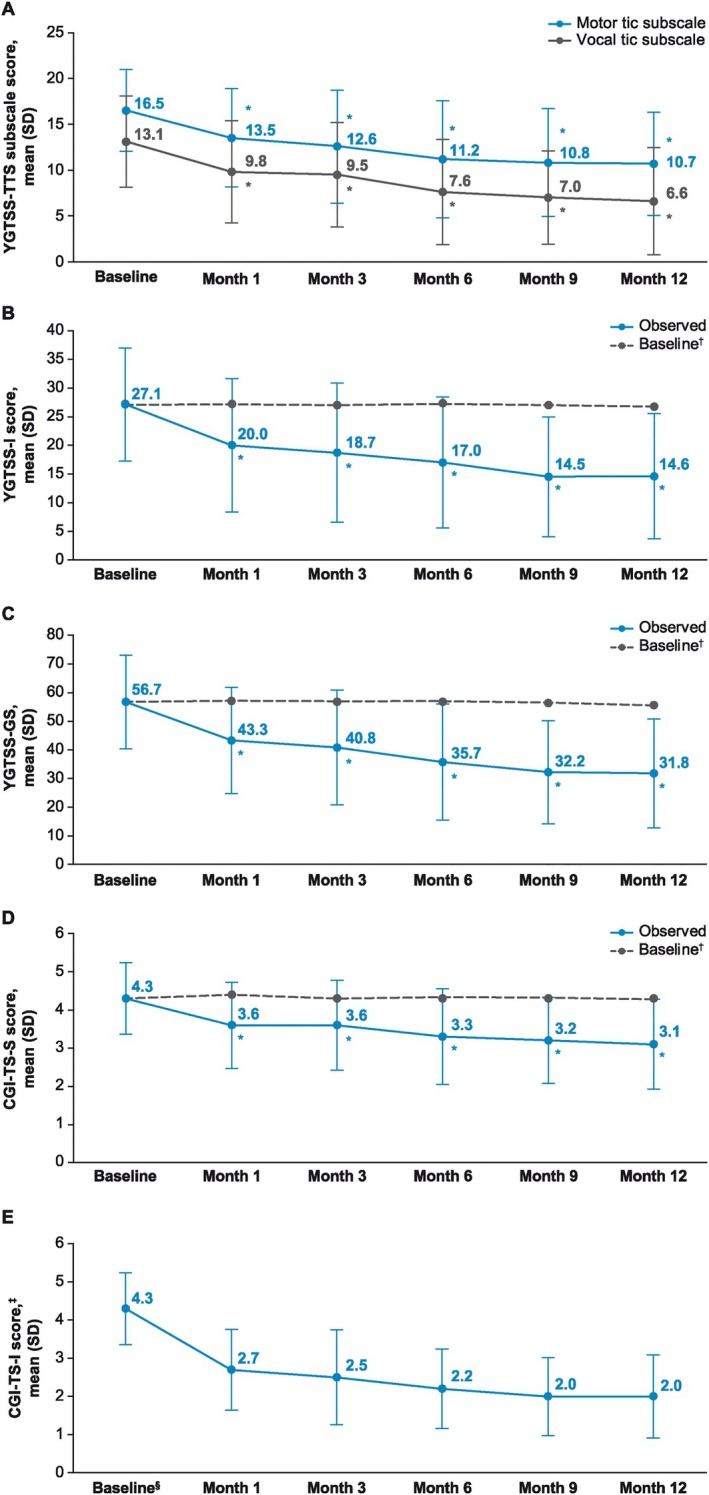
TS severity and improvement over time using (**A**) YGTSS‐TTS motor and vocal tic subscales. (**B**) YGTSS‐I. (**C**) YGTSS‐GS. (**D**) CGI‐TS‐S. (**E**) CGI‐TS‐I, with lower scores indicating improvement. *P < 0.0001 vs. baseline. ^†^Baseline for patients with data at that time point. ^‡^Range from 1 (“very much improved”) to 7 (“very much worse”). ^§^CGI‐TS‐S baseline. CGI‐TS‐I, Clinical Global Impression of Tourette Syndrome Improvement; CGI‐TS‐S, Clinical Global Impression of Tourette Syndrome Severity; SD, standard deviation; TS, Tourette syndrome; YGTSS‐GS, Yale Global Tic Severity Scale Global Score; YGTSS‐I, Yale Global Tic Severity Scale Impairment; YGTSS‐TTS, Yale Global Tic Severity Scale Total Tic Score.

**FIG. 4:**
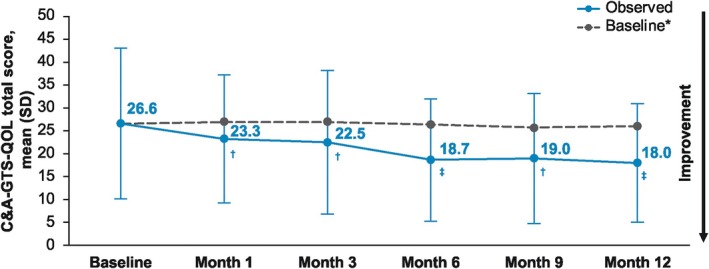
C&A‐GTS‐QOL score over time, with lower scores indicating improvement. *Baseline for patients with data at that time point. ^†^ *P* < 0.001 vs. baseline. ^‡^ *P* < 0.0001 vs. baseline. C&A‐GTS‐QOL, Gilles de la Tourette Syndrome Quality of Life Scale for Children and Adolescents; SD, standard deviation.

A significant improvement from OLE baseline in YGTSS‐TTS was observed at all time points (*P* < 0.0001 for all) (Fig. 2A). A threshold ≥25% reduction in YGTSS‐TTS score (ie, percentage reduction in YGTSS‐TTS corresponding with a positive change on the CGI‐TS‐I^17^) was met from month 3 onward, with a 40.3% mean reduction in YGTSS‐TTS at month 12 (mean [SD] change, −11.7 [9.5]). Significantly improved YGTSS‐TTS scores were observed at all time points regardless of treatment arm assigned in the phase 2b randomized controlled trial (*P* < 0.0001 vs baseline for all time points and groups) (Fig. 2B). Significant mean improvements from baseline through month 12 were also observed for the YGTSS‐TTS motor and vocal subscales (Fig. 3A), YGTSS‐I (Fig. 3B), YGTSS‐GS (Fig. 3C), and CGI‐TS‐S (Fig. 3D) (*P* < 0.0001 vs. baseline for all time points). Mean CGI‐TS‐I scores through month 12 indicated TS improvement from baseline (Fig. 3E). In addition, significant mean improvements from baseline were observed for the C&A‐GTS‐QOL total score through month 12 (*P* < 0.001 for all time points; Fig. 4). At month 12, the mean (SD) change from baseline in C&A‐GTS‐QOL score was −8.0 (16.2).

## Discussion

This OLE study demonstrated a favorable safety and tolerability profile for ecopipam 1.8 mg/kg/day as well as reduced tic severity and improved QOL during 12 months of treatment in children and adolescents with TS. Ecopipam treatment was not associated with significant increases in BMI Z‐score, metabolic abnormalities, changes in ECG measures, or drug‐induced movement disorders. These results are consistent with the efficacy and safety profile of ecopipam for TS reported in a phase 2b randomized, double‐blind, placebo‐controlled trial in which this pediatric patient population had previously participated,^16^ as well as data from a phase 2b, 4‐week randomized, double‐blind, placebo‐controlled crossover study of children and adolescents with TS^18^ and its OLE.^19^

The safety profile of ecopipam is particularly important when considered in comparison with the only three medications approved in the United States for TS: haloperidol, pimozide, and aripiprazole. These three antipsychotics, all of which act at dopamine D2 receptors (D2Rs), are effective for tic reduction but have greater metabolic, cardiac, and neurological risks.^8^, ^9^, ^10^, ^11^ For example, a meta‐analysis (60 randomized trials) of 4077 patients with tic disorders estimated the prevalence of drug‐induced movement disorders to be 6.4% to 19.4% for aripiprazole and 40% to 43.6% for haloperidol.^20^ The estimated prevalence rates of akathisia, slowed reaction, and postural stiffness with pimozide were 10% to 40%, 5% to 10%, and 20%, respectively.^20^ Movement disorders can impair QOL, as seen in a study of patients taking antipsychotics for treatment of schizophrenia, where fewer extrapyramidal symptoms significantly predicted higher QOL.^21^ Additionally, in a study of pediatric patients with no previous antipsychotic use, 12 weeks of treatment with aripiprazole (n = 41) led to significant increases from baseline in BMI (mean change from baseline, 1.7 kg/m^2^ [7.2%]) and BMI Z‐score (0.37 points) (*P* < 0.001 for both), and 7.3% of patients developed insulin resistance.^22^ To date, there is no evidence for these AEs in studies of ecopipam. Taken together, the AEs documented in the randomized controlled trials and OLE studies suggest that ecopipam, from a safety standpoint, is more similar to the α2 receptor agonists guanfacine and clonidine than it is to the antipsychotics.

In the current study, ecopipam significantly reduced TS symptom severity from baseline through 12 months of treatment, with no evidence of tachyphylaxis. A ≥25% improvement in YGTSS‐TTS score was achieved at month 3 and sustained through month 12, with a 40.3% mean reduction from baseline after 12 months of ecopipam treatment. It is possible that this threshold for clinical meaningfulness was not met at month 1 because the ecopipam dose was titrated to the maintenance dose over the first 4 weeks of treatment. These results are consistent with those from the randomized trial,^16^ where ecopipam led to a mean reduction of 30% in YGTSS‐TTS from baseline to week 12 (*P* = 0.01 vs. placebo), and support the continued benefit of long‐term ecopipam treatment. Previous treatment exposure did not appear to influence the effect of ecopipam in the OLE, as significant improvements from baseline in symptoms and QOL measures were observed regardless of whether patients had been treated with ecopipam or placebo during the phase 2b randomized trial.

There is strong evidence of a relationship among TS symptom severity, associated comorbidities, and QOL impairment. A systematic review of 13 studies assessing health‐related QOL in patients with TS concluded that tic severity and psychiatric comorbidities, particularly ADHD and OCD, are associated with poorer health‐related QOL in children.^23^ Diminished QOL in patients with TS is observed across physical, cognitive, work or school, emotional, and social domains.^5^ The C&A‐GTS‐QOL is a validated, tic disorder–specific, 27‐item questionnaire that assesses QOL across cognitive, coprophenomena, psychological, physical, obsessive‐compulsive, and activities of daily living domains.^24^ In the current study, ecopipam 1.8 mg/kg/day significantly improved C&A‐GTS‐QOL scores through 12 months of treatment, which suggests that the clinical benefits of long‐term ecopipam treatment extend beyond symptom severity reduction to include improvement in QOL in children and adolescents with TS.

Excess dopaminergic activity in the central nervous system plays a key role in the pathophysiology of TS.^25^ Currently approved therapies for TS are antipsychotics with antagonist activity at D2Rs, which are primarily expressed on medium spiny neurons in the indirect pathway of the basal ganglia.^10^, ^26^ In contrast, D1Rs are primarily expressed in the direct pathway.^26^ Under physiologic conditions, dopaminergic signaling in these pathways coordinates the selection and initiation of voluntary actions (direct pathway) and suppression of unwanted or involuntary actions (indirect pathway).^25^, ^26^, ^27^ In TS, excess dopamine may cause imbalanced activation of these pathways, leading to inappropriate movements (ie, tics).^25^ Results from this OLE and phase 2b trials suggest that the selective D1R antagonist ecopipam reduces tic severity while circumventing drug‐induced movement disorders associated with D2R antagonists.

This is the largest study to date to examine the long‐term safety and effectiveness of ecopipam in a pediatric population with TS. Data are encouraging and showed that 12‐month treatment with ecopipam reduced TS symptom severity and improved QOL without many of the adverse effects associated with currently approved agents for TS.^8^, ^9^, ^10^, ^11^, ^28^ Limitations of this study include the lack of a control arm, exclusion of adults with TS, and limited racial and ethnic diversity. In addition, only one‐third of patients enrolled in the trial were age 6 to 11 years. There are several possible reasons for the lower representation in this younger age group. First, although tic severity, as scored by the YGTSS, tends to peak at approximately 8 to 12 years of age,^29^ stigma and functional impairment can be higher in adolescence, thereby enhancing motivation for participation in older individuals. Second, younger patients often have not yet had pharmacologic treatment for TS, and parents or caregivers and primary health care providers may prefer to use recommended first‐line treatments before considering investigational medications. Regarding representation of common comorbid conditions, only 14.9% of patients in this study had a history of OCD, whereas the lifetime prevalence rate of OCD in patients with TS has been estimated at 66.1%.^3^ Therefore, there was limited opportunity to evaluate the impact of treatment on tics and OCD symptoms in this population. As well, this OLE study included only patients who had completed the phase 2b, randomized, double‐blind, placebo‐controlled trial.^16^ Therefore, there was potential for bias because patients who had previously received ecopipam and had a positive experience may have been more likely to enroll in the OLE, whereas those with tolerability issues or lack of efficacy may have been less likely to enroll. However, all patients who completed the phase 2b trial, regardless of treatment response, could enter the OLE. Additionally, 124 of 133 patients (93.2%) who completed the phase 2b trial enrolled in the OLE, and no substantial differences in demographics, tic severity (YGTSS‐TTS), psychiatric comorbidities, or weight at OLE baseline between the phase 2b treatment arms were noted, suggesting low risk for selection bias.

In conclusion, daily ecopipam appeared to be well tolerated and safe during 12 months of treatment, with tic severity and QOL improvements observed over the long term. Ecopipam may be a safe and effective alternative to currently available pharmacotherapies for treatment of tics in pediatric patients with TS. Further research is warranted, and a phase 3 trial of ecopipam for the treatment of TS in patients age ≥6 years has been completed (NCT05615220).

## Author Roles

(1) Research Project: A. Conception, B. Organization, C. Execution; (2) Statistical Analysis: A. Design, B. Execution, C. Review and Critique; (3) Manuscript Preparation: A. Writing of the First Draft, B. Review and Critique.

D.L.G.: 1A, 1C, 2C, 3B

D.J.B.K.: 1A, 1C, 2C, 3B

M.M.M.: 1A, 1C, 2C, 3B

S.D.A.: 1A, 1C, 2C, 3B

G.B.K.: 1A, 1C, 2C, 3A, 3B

F.E.M.: 1A, 1C, 2C, 3B

S.P.W.: 1A, 1C, 2C, 3B

T.M.C.: 1A, 1C, 2C, 3B

## Disclosures

**Ethical Compliance Statement**: The study was conducted in accordance with the International Council on Harmonization of Good Clinical Practice and the ethical principles of the Declaration of Helsinki. The study protocol, including the informed consent and assent forms, received written institutional review board approval (Advarra, Columbia, MD; IRB00000971). For patients <18 years of age, parents or legal guardians provided written informed consent and patients provided written assent before the initiation of any study procedures. Patients 18 years of age provided written informed consent. We confirm that we have read the Journal's position on issues involved in ethical publication and affirm that this work is consistent with those guidelines.

**Funding Sources and Conflict of Interest**: D.L.G. reports being a clinical trial site investigator for Emalex Biosciences. D.J.B.K., M.M.M., S.D.A., G.B.K., and F.E.M. are employees of and have personal equity interest in Emalex Biosciences. S.P.W. and T.M.C. are employees of Paragon Biosciences, which has controlling equity interest in Emalex Biosciences, and have personal equity interest in Emalex Biosciences.

**Financial Disclosures for the Previous 12 Months**: D.L.G. reports being a clinical trial site investigator for PTC Therapeutics. D.J.B.K., M.M.M., S.D.A., G.B.K., F.E.M., S.P.W., and T.M.C. declare that there are no additional disclosures to report. Dr. Gilbert has received compensation for expert testimony for the U.S. National Vaccine Injury Compensation Program, through the Department of Health and Human Services. He has received payment for medical expert opinions through TeladocHealth International. He has served as a paid consultant for PTC Therapeutics, Noema Pharma, and Emalex Biosciences and has received travel support to attend investigator meetings. He has provided educational lectures for Illumina, Inc and PTC Therapeutics, Inc. He has received research support from the United States National Institutes of Health (Tourette Syndrome, ADHD research) and the Department of Defense (Neurofibromatosis research). He has received salary compensation through Cincinnati Children’s for work as a clinical trial site investigator from Emalex (clinical trial, Tourette Syndrome), PTC Therapeutics (registry and clinical trial, Amino Acid Decarboxylase Deficiency), and Quince Therapeutics (clinical trial, ataxia telangiectasia). He has received book/publication royalties from Elsevier and Wolters Kluwer.

## Supporting information


**Data S1** **Supplemental Methods**. Listing of Adverse Events of Special Interest.
**Table S1.** Patient Disposition.
**Table S2.** AE‐Related Study Discontinuations During OLE Study.
**Table S3.** AEs of Special Interest.
**Table S4.** Additional Safety Outcomes.

## Data Availability

The data that support the findings of this trial are available on reasonable written request.
