# Supplementary material for: Safety and Effect of 12‐Month Ecopipam Treatment in Pediatric Patients with Tourette Syndrome
Source: Mov Disord Clin Pract. 2025 May 12;12(8):1157–66. doi: 10.1002/mdc3.70091 (PMC12371453; doi:10.1002/mdc3.70091)
Supplement: Supplementary file 1 — Data S1 Supplemental Methods. Listing of Adverse Events of Special Interest. Table S1. Patient Disposition. Table S2. AE‐Related Study Discontinuations During OLE Study. Table S3. AEs of Special Interest. Table S4. Additional Safety Outcomes. [file MDC3-12-1157-s001.docx]

**Safety and Effect of 12-Month Ecopipam Treatment in Pediatric Patients With Tourette Syndrome**

Donald L. Gilbert, MD, MS^1^; David J.B. Kim, BS^2^; Meredith M. Miller, BS^2^; Sarah D. Atkinson, MD^2^; George B. Karkanias, PhD, MBA, MS^2^; Frederick E. Munschauer, MD^2^; Stephen P. Wanaski, PhD^3^; Timothy M. Cunniff, PharmD^3^

^1^Cincinnati Children’s Hospital Medical Center, University of Cincinnati College of Medicine, Cincinnati, OH; ^2^Emalex Biosciences, Inc., Chicago, IL; ^3^Paragon Biosciences, LLC, Chicago, IL

**SUPPLEMENTAL MATERIALS**

**Supplemental Methods.** Listing of Adverse Events of Special Interest

Adverse events of special interest were: agitation, amnesia, anosmia, anxiety, apathy, ataxia, balance disorder, bradykinesia, confusional state, constipation, convulsions, coordination abnormal, decreased weight, dementia, dependence and withdrawal, depression or related terms (depression, depressed mood, depressive symptom, and major depression), disturbance in attention, dizziness, drooling, drug abuse, dysgraphia, dyskinesia, dyslipidemia, dysphagia, dysphonia, dysstasia, dystonia, fall, fatigue, fear of falling, flat affect, gait disturbance, hepatic disorders, hyperglycemia, hyperkinesia, joint stiffness, loss of visual contrast sensitivity, memory impairment, mental impairment, movement disorder, muscle contracture, muscle rigidity, muscle tightness, musculoskeletal stiffness, neuroleptic malignant syndrome, new-onset diabetes mellitus, nightmare, parosmia, poor-quality sleep, posture abnormal, psychosis and psychotic disorders, reduced facial expression, restlessness, self-injury, sleep disorder, somnolence, speech disorder, suicide, terminal insomnia, tremor, urinary incontinence, vertigo, and vocal cord dysfunction.

**Supplemental Table 1.** Patient Disposition

| **Population** | **Patients, n (%)** |
| --- | --- |
| Enrolled | 124 |
| Safety population^*^ | 121 (97.6) |
| Modified intent-to-treat population^†^ | 121 (97.6) |
| Completed study | 80 (64.5) |
| Discontinued prematurely  Withdrawal of consent/withdrawal by parent/caregiver  Adverse event  Other  Investigator decision  Lost to follow-up  Protocol deviation  Treatment nonadherence | 44 (35.5) 18 (14.5) 14 (11.3) 5 (4.0) 2 (1.6) 2 (1.6) 2 (1.6) 1 (0.8) |

^*^Received ≥1 dose of study drug.

^†^Received ≥1 dose of study drug and had ≥1 postbaseline Yale Global Tic Severity Scale score.

**Supplemental Table 2.** AE-Related Study Discontinuations During OLE Study

| **AE leading to discontinuation** | **Patients who discontinued due to AE, n (%) (n=14)** |
| --- | --- |
| Depression^*^ | 4 (28.6) |
| Anxiety | 2 (14.3) |
| Abdominal discomfort | 1 (7.1) |
| Aggression | 1 (7.1) |
| Akathisia | 1 (7.1) |
| Decreased appetite | 1 (7.1) |
| Diarrhea | 1 (7.1) |
| Headache | 1 (7.1) |
| Insomnia | 1 (7.1) |
| Joint lock | 1 (7.1) |
| Mental disorder | 1 (7.1) |
| Migraine | 1 (7.1) |
| Obsessive thoughts | 1 (7.1) |
| Somnolence | 1 (7.1) |
| Upper abdominal pain | 1 (7.1) |

^*^Included depression, depressive mood, and depressive symptom.

AE = adverse event; OLE = open-label extension.

**Supplemental Table 3.** AEs of Special Interest

| **AE of special interest** | **Patients, n (%) (n=121)** |
| --- | --- |
| Any | 24 (19.8) |
| Treatment-related | 14 (11.6) |
| Occurring in ≥2 patients  Depression^*^  Pyrexia  Suicidal ideation  Tremor  Weight decreased | 9 (7.4) 7 (5.8) 3 (2.5) 2 (1.7) 2 (1.7) |

^*^Included depression, depressed mood, depressive symptom, and major depression.
AE = adverse event.

**Supplemental Table 4.** Additional Safety Outcomes

| **Parameter** | | **OLE Baseline** | **Month** | | | | | | | | | | | |
| --- | --- | --- | --- | --- | --- | --- | --- | --- | --- | --- | --- | --- | --- | --- |
|  |  |  | **1** | **2** | **3** | **4** | **5** | **6** | **7** | **8** | **9** | **10** | **11** | **12** |
| AIMS total score^*^ | Pt, n | 121 | 116 | 112 | 107 | 102 | 98 | 94 | 85 | 88 | 89 | 81 | 80 | 80 |
|  | Mean (SD) | 5.4 (9.1) | 4.4 (7.0) | 4.6 (7.5) | 4.1 (6.6) | 3.7 (6.5) | 3.9 (6.3) | 3.7 (6.2) | 3.6 (5.3) | 4.0 (6.0) | 4.0 (6.1) | 4.1 (6.2) | 3.6 (5.2) | 3.6 (5.3) |
|  | Range | 0-32 | 0-28 | 0-31 | 0-27 | 0-25 | 0-23 | 0-29 | 0-21 | 0-26 | 0-25 | 0-29 | 0-23 | 0-19 |
| BARS objective akathisia^†^ | Pt, n | 121 | 116 | 112 | 107 | 102 | 98 | 94 | 85 | 88 | 89 | 81 | 80 | 80 |
|  | Mean (SD) | 0.2 (0.6) | 0.2 (0.5) | 0.1 (0.4) | 0.1 (0.4) | 0.1 (0.4) | 0.1 (0.4) | 0.1 (0.4) | 0.1 (0.4) | 0.1 (0.4) | 0.2 (0.4) | 0.1 (0.4) | 0.1 (0.4) | 0.1 (0.4) |
|  | Range | 0-3 | 0-2 | 0-2 | 0-2 | 0-2 | 0-2 | 0-2 | 0-2 | 0-2 | 0-2 | 0-2 | 0-2 | 0-2 |
| BARS subjective awareness of restlessness^†^ | Pt, n | 121 | 116 | 112 | 107 | 102 | 98 | 94 | 85 | 88 | 89 | 81 | 80 | 80 |
|  | Mean (SD) | 0.3 (0.6) | 0.3 (0.6) | 0.3 (0.6) | 0.2 (0.5) | 0.1 (0.4) | 0.1 (0.4) | 0.2 (0.5) | 0.2 (0.5) | 0.2 (0.5) | 0.2 (0.4) | 0.2 (0.5) | 0.2 (0.5) | 0.2 (0.5) |
|  | Range | 0-2 | 0-2 | 0-2 | 0-2 | 0-2 | 0-2 | 0-2 | 0-2 | 0-2 | 0-2 | 0-2 | 0-2 | 0-2 |
| Subjective distress related to restlessness^†^ | Pt, n | 120 | 115 | 112 | 106 | 101 | 98 | 93 | 85 | 87 | 89 | 81 | 80 | 80 |
|  | Mean (SD) | 0.2 (0.5) | 0.2 (0.5) | 0.2 (0.5) | 0.1 (0.4) | 0.1 (0.3) | 0.1 (0.3) | 0.1 (0.3) | 0.1 (0.3) | 0.1 (0.3) | 0.1 (0.3) | 0.1 (0.4) | 0.1 (0.3) | 0.1 (0.4) |
|  | Range | 0-2 | 0-2 | 0-2 | 0-2 | 0-2 | 0-2 | 0-2 | 0-2 | 0-2 | 0-2 | 0-2 | 0-1 | 0-2 |
| BARS total score^‡^ | Pt, n | 121 | 116 | 112 | 107 | 102 | 98 | 94 | 85 | 88 | 89 | 81 | 80 | 80 |
|  | Mean (SD) | 0.7 (1.6) | 0.7 (1.4) | 0.6 (1.4) | 0.5 (1.2) | 0.3 (1.1) | 0.3 (1.0) | 0.4 (1.1) | 0.4 (1.2) | 0.4 (1.1) | 0.4 (1.1) | 0.4 (1.1) | 0.4 (1.1) | 0.4 (1.2) |
|  | Range | 0-7 | 0-6 | 0-6 | 0-6 | 0-6 | 0-6 | 0-6 | 0-6 | 0-6 | 0-6 | 0-6 | 0-5 | 0-5 |
| BARS global clinical assessment of akathisia^§^ | Pt, n | 121 | 116 | 112 | 107 | 102 | 98 | 94 | 85 | 88 | 89 | 81 | 80 | 80 |
|  | Mean (SD) | 0.3 (0.8) | 0.3 (0.7) | 0.3 (0.7) | 0.2 (0.7) | 0.2 (0.6) | 0.1 (0.5) | 0.2 (0.6) | 0.2 (0.6) | 0.2 (0.6) | 0.2 (0.6) | 0.2 (0.6) | 0.2 (0.6) | 0.3 (0.7) |
|  | Range | 0-3 | 0-3 | 0-3 | 0-3 | 0-3 | 0-2 | 0-3 | 0-2 | 0-3 | 0-3 | 0-2 | 0-2 | 0-3 |
| CDRS-R^¶^ | Pt, n | 121 | 119 | 113 | 110 | 102 | 98 | 94 | 89 | 89 | 89 | 82 | 80 | 80 |
|  | Mean (SD) | 26.4 (8.7) | 24.8 (8.1) | 24.7 (8.2) | 24.7 (8.6) | 23.7 (6.6) | 24.0 (6.9) | 23.5 (7.0) | 23.9 (7.2) | 24.2 (7.4) | 24.3 (7.9) | 23.7 (7.9) | 23.8 (8.1) | 24.0 (8.6) |
|  | Range | 16-58 | 17-55 | 16-63 | 17-59 | 16-44 | 16-42 | 17-48 | 17-48 | 17-52 | 17-48 | 17-50 | 17-54 | 16-68 |
| PARS^#,**^ | Pt, n | 119 | 118 | 111 | 107 | 98 | 97 | 94 | 88 | 88 | 88 | 82 | 80 | 80 |
|  | Mean (SD) | 9.0 (7.0) | 7.9 (6.9) | 8.6 (7.4) | 8.7 (7.4) | 7.5 (7.2) | 8.0 (7.3) | 7.8 (7.2) | 7.7 (7.0) | 8.2 (7.7) | 8.4 (7.7) | 7.2 (7.3) | 7.0 (7.6) | 7.5 (7.4) |
|  | Range | 0-28 | 0-31 | 0-31 | 0-29 | 0-27 | 0-27 | 0-27 | 0-29 | 0-27 | 0-27 | 0-26 | 0-27 | 0-26 |

AIMS = Abnormal Involuntary Movement Scale; BARS = Barnes Akathisia Rating Scale; CDRS-R = Children’s Depression Rating Scale-Revised; OLE = open-label extension; PARS = Pediatric Anxiety Rating Scale; Pt = patients.

^*^Total score is calculated by adding the scores for each of the 10 items, each of which is rated on a scale from 0 (none) to 4 (severe), for a total score ranging from 0 to 40.

^†^Rated on a scale from 0 (normal) to 3 (most severe).

^‡^Total score is calculated by adding the scores for each of 3 items (objective akathisia, subjective awareness of restlessness, and subjective distress related to restlessness), each of which is rated on a scale from 0 (normal) to 3 (most severe), for a total score ranging from 0 to 9.

^§^Rated on a scale from 0 (absent) to 5 (severe).

^¶^Total score is calculated by adding the scores for 14 psychiatric signs and symptoms of depression on a scale from 1 (normal) to 7 (most severe) and 3 signs and symptoms on a scale from 1 (normal) to 5 (most severe), for a total score ranging from 17 to 113 (if no records are missing).

^#^One patient was excluded from the analysis due to an error in translation of the French Canadian PARS.

^**^Total score is calculated by adding the scores for the 7 items rated on a scale from 0 (none) to 5 (most extreme), for a total score ranging from 0 to 35.
